# (4-Bromo-3,5-dimethyl-1*H*-pyrazol-1-yl)(2,6-difluoro­phen­yl)methanone

**DOI:** 10.1107/S1600536809052532

**Published:** 2009-12-12

**Authors:** Xiang-Dong Mei, Yan-Hui Liang, Zhong-Yue Wang

**Affiliations:** a Key Laboratory of Pesticide Chemistry and Applications, Ministry of Agriculture, Institute of Plant Protection, Chinese Academy of Agricultural Sciences, Beijing 100193, People’s Republic of China

## Abstract

There are two mol­ecules in the asymmetric unit of the title compound, C_12_H_9_BrF_2_N_2_O. They have very similar conformations: the dihedral angles between their pyrazole and benzene ring systems are 78.4 (3) and 78.6 (4)°. In the crystal, weak aromatic π–π stacking [centroid–centroid separation = 3.696 (5) Å] helps to establish the packing.

## Related literature

For background to pyrazole derivatives in agrochemical and medicinal research, see: Sabbagh *et al.* (2009 ▶); Zheng *et al.* (2009 ▶).
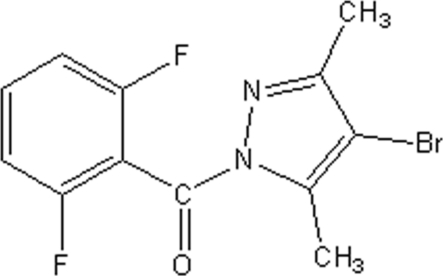


## Experimental

### 

#### Crystal data

C_12_H_9_BrF_2_N_2_O
*M* *_r_* = 315.12
Monoclinic, 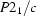

*a* = 7.116 (3) Å
*b* = 29.304 (10) Å
*c* = 11.674 (4) Å
β = 91.533 (5)°
*V* = 2433.5 (15) Å^3^
*Z* = 8
Mo *K*α radiation
μ = 3.39 mm^−1^
*T* = 173 K
0.17 × 0.17 × 0.17 mm

#### Data collection

Rigaku Saturn724+ CCD diffractometer
Absorption correction: numerical (*CrystalClear*; Rigaku, 2008 ▶) *T*_min_ = 0.596, *T*_max_ = 0.596
15188 measured reflections
4276 independent reflections
3819 reflections with *I* > 2σ(*I*)
*R*_int_ = 0.060

#### Refinement

*R*[*F* ^2^ > 2σ(*F* ^2^)] = 0.077
*wR*(*F* ^2^) = 0.158
*S* = 1.27
4276 reflections
329 parameters
H-atom parameters constrained
Δρ_max_ = 1.08 e Å^−3^
Δρ_min_ = −0.47 e Å^−3^

### 

Data collection: *CrystalClear* (Rigaku, 2008 ▶); cell refinement: *CrystalClear*; data reduction: *CrystalClear*; program(s) used to solve structure: *SHELXS97* (Sheldrick, 2008 ▶); program(s) used to refine structure: *SHELXL97* (Sheldrick, 2008 ▶); molecular graphics: *SHELXTL* (Sheldrick, 2008 ▶); software used to prepare material for publication: *SHELXL97*.

## Supplementary Material

Crystal structure: contains datablocks I, global. DOI: 10.1107/S1600536809052532/hb5260sup1.cif

Structure factors: contains datablocks I. DOI: 10.1107/S1600536809052532/hb5260Isup2.hkl

Additional supplementary materials:  crystallographic information; 3D view; checkCIF report

## Figures and Tables

**Fig. 1.**
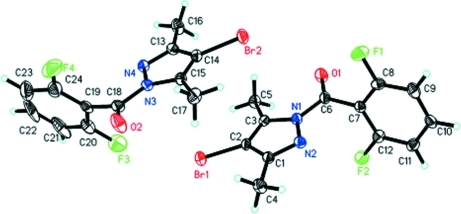
The molecular structure of the title compound showing 50% probability displacement ellipsoids and atom-numbering scheme.

## supplementary crystallographic information

### Experimental

The title compound (0.2 g) was dissolved in ethanol (50 ml) at room temperature. Colourless crystals of compound (I) were obtained through slow evaporation after two weeks.

### Refinement

The H atoms were placed at calculated positions, with C—H ═ 0.93–0.98 Å, and refined as riding with *U*iso(H) = 1.2–1.5*U*ep(C).

### Crystal data

**Table:**

C_12_H_9_BrF_2_N_2_O	*F*(000) = 1248
*M**_r_* = 315.12	*D*_x_ = 1.720 Mg m^−^^3^
Monoclinic, *P*2_1_/*c*	Mo *K*α radiation, λ = 0.71073 Å
Hall symbol: -P 2ybc	Cell parameters from 768 reflections
*a* = 7.116 (3) Å	θ = 2.2–27.5°
*b* = 29.304 (10) Å	µ = 3.39 mm^−^^1^
*c* = 11.674 (4) Å	*T* = 173 K
β = 91.533 (5)°	Block, colourless
*V* = 2433.5 (15) Å^3^	0.17 × 0.17 × 0.17 mm
*Z* = 8	

### Data collection

**Table:**

Rigaku Saturn724+ CCD diffractometer	4276 independent reflections
Radiation source: sealed tube	3819 reflections with *I* > 2σ(*I*)
graphite	*R*_int_ = 0.060
ω scans at fixed χ = 45°	θ_max_ = 25.0°, θ_min_ = 2.8°
Absorption correction: numerical (*CrystalClear*; Rigaku, 2008)	*h* = −6→8
*T*_min_ = 0.596, *T*_max_ = 0.596	*k* = −34→34
15188 measured reflections	*l* = −12→13

### Refinement

**Table:**

Refinement on *F*^2^	Primary atom site location: structure-invariant direct methods
Least-squares matrix: full	Secondary atom site location: difference Fourier map
*R*[*F*^2^ > 2σ(*F*^2^)] = 0.077	Hydrogen site location: inferred from neighbouring sites
*wR*(*F*^2^) = 0.158	H-atom parameters constrained
*S* = 1.27	*w* = 1/[σ^2^(*F*_o_^2^) + (0.0402*P*)^2^ + 7.5951*P*] where *P* = (*F*_o_^2^ + 2*F*_c_^2^)/3
4276 reflections	(Δ/σ)_max_ < 0.001
329 parameters	Δρ_max_ = 1.08 e Å^−^^3^
0 restraints	Δρ_min_ = −0.47 e Å^−^^3^

### Special details

**Table:**

Geometry. All e.s.d.'s (except the e.s.d. in the dihedral angle between two l.s. planes) are estimated using the full covariance matrix. The cell e.s.d.'s are taken into account individually in the estimation of e.s.d.'s in distances, angles and torsion angles; correlations between e.s.d.'s in cell parameters are only used when they are defined by crystal symmetry. An approximate (isotropic) treatment of cell e.s.d.'s is used for estimating e.s.d.'s involving l.s. planes.
Refinement. Refinement of *F*^2^ against ALL reflections. The weighted *R*-factor *wR* and goodness of fit *S* are based on *F*^2^, conventional *R*-factors *R* are based on *F*, with *F* set to zero for negative *F*^2^. The threshold expression of *F*^2^ > σ(*F*^2^) is used only for calculating *R*-factors(gt) *etc*. and is not relevant to the choice of reflections for refinement. *R*-factors based on *F*^2^ are statistically about twice as large as those based on *F*, and *R*- factors based on ALL data will be even larger.

### Fractional atomic coordinates and isotropic or equivalent isotropic displacement parameters (Å^2^)

**Table:**

	*x*	*y*	*z*	*U*_iso_*/*U*_eq_	
Br1	0.48772 (10)	0.41014 (2)	0.89703 (6)	0.0409 (2)	
Br2	1.00200 (10)	0.31950 (3)	1.09739 (6)	0.0491 (3)	
F1	0.7911 (6)	0.30667 (16)	1.4485 (4)	0.0586 (12)	
F2	0.1573 (5)	0.32948 (15)	1.3484 (3)	0.0503 (11)	
F3	0.6692 (7)	0.42324 (19)	0.6390 (5)	0.0780 (16)	
F4	1.3057 (7)	0.4580 (2)	0.6345 (6)	0.0889 (18)	
O1	0.5532 (8)	0.26916 (16)	1.2410 (4)	0.0534 (14)	
O2	1.0003 (10)	0.48031 (17)	0.8138 (4)	0.0649 (17)	
N1	0.5031 (7)	0.34346 (17)	1.1966 (4)	0.0298 (12)	
N2	0.4946 (7)	0.38788 (17)	1.2406 (5)	0.0322 (12)	
N3	0.9873 (7)	0.40262 (16)	0.8255 (4)	0.0290 (12)	
N4	0.9923 (7)	0.36189 (17)	0.7664 (4)	0.0303 (12)	
C1	0.4918 (9)	0.4144 (2)	1.1501 (6)	0.0323 (15)	
C2	0.4947 (9)	0.3874 (2)	1.0474 (5)	0.0304 (14)	
C3	0.5023 (8)	0.3431 (2)	1.0775 (5)	0.0287 (14)	
C4	0.4855 (11)	0.4650 (2)	1.1623 (6)	0.0431 (17)	
H4A	0.4898	0.4731	1.2438	0.065*	
H4B	0.3690	0.4767	1.1265	0.065*	
H4C	0.5936	0.4785	1.1247	0.065*	
C5	0.5039 (10)	0.3009 (2)	1.0060 (6)	0.0380 (16)	
H5A	0.4885	0.3092	0.9250	0.057*	
H5B	0.4005	0.2809	1.0279	0.057*	
H5C	0.6238	0.2850	1.0183	0.057*	
C6	0.5157 (10)	0.3064 (2)	1.2725 (5)	0.0351 (15)	
C7	0.4758 (9)	0.3177 (2)	1.3954 (5)	0.0302 (14)	
C8	0.6131 (9)	0.3157 (2)	1.4813 (6)	0.0362 (16)	
C9	0.5761 (11)	0.3226 (2)	1.5946 (6)	0.0434 (18)	
H9A	0.6742	0.3219	1.6513	0.052*	
C10	0.3931 (10)	0.3306 (2)	1.6239 (6)	0.0387 (16)	
H10A	0.3650	0.3341	1.7025	0.046*	
C11	0.2501 (10)	0.3337 (2)	1.5433 (6)	0.0409 (17)	
H11A	0.1250	0.3405	1.5642	0.049*	
C12	0.2950 (9)	0.3266 (2)	1.4315 (6)	0.0355 (15)	
C13	0.9966 (9)	0.3303 (2)	0.8459 (6)	0.0330 (15)	
C14	0.9948 (9)	0.3504 (2)	0.9562 (5)	0.0325 (15)	
C15	0.9901 (9)	0.3960 (2)	0.9434 (5)	0.0339 (15)	
C16	1.0047 (12)	0.2810 (2)	0.8158 (7)	0.051 (2)	
H16A	1.0217	0.2777	0.7332	0.076*	
H16B	0.8872	0.2661	0.8369	0.076*	
H16C	1.1105	0.2666	0.8575	0.076*	
C17	0.9875 (11)	0.4331 (3)	1.0304 (6)	0.0481 (19)	
H17A	0.9903	0.4197	1.1074	0.072*	
H17B	0.8729	0.4512	1.0195	0.072*	
H17C	1.0977	0.4527	1.0217	0.072*	
C18	0.9919 (10)	0.4439 (2)	0.7649 (6)	0.0378 (16)	
C19	0.9876 (10)	0.4396 (2)	0.6374 (6)	0.0374 (16)	
C20	0.8243 (12)	0.4300 (3)	0.5776 (7)	0.052 (2)	
C21	0.8162 (19)	0.4272 (3)	0.4604 (8)	0.087 (4)	
H21A	0.7018	0.4200	0.4206	0.105*	
C22	0.975 (2)	0.4350 (3)	0.4030 (8)	0.100 (5)	
H22A	0.9711	0.4331	0.3217	0.120*	
C23	1.141 (2)	0.4456 (4)	0.4571 (10)	0.090 (4)	
H23A	1.2508	0.4512	0.4148	0.108*	
C24	1.1454 (13)	0.4478 (3)	0.5754 (7)	0.058 (2)	

### Atomic displacement parameters (Å^2^)

**Table:**

	*U*^11^	*U*^22^	*U*^33^	*U*^12^	*U*^13^	*U*^23^
Br1	0.0507 (5)	0.0423 (4)	0.0296 (4)	−0.0035 (3)	0.0003 (3)	0.0051 (3)
Br2	0.0450 (4)	0.0693 (6)	0.0328 (4)	−0.0021 (4)	−0.0021 (3)	0.0171 (4)
F1	0.036 (2)	0.073 (3)	0.067 (3)	0.004 (2)	0.001 (2)	−0.013 (2)
F2	0.039 (2)	0.071 (3)	0.041 (2)	0.006 (2)	−0.0080 (19)	−0.004 (2)
F3	0.055 (3)	0.076 (4)	0.102 (4)	−0.014 (3)	−0.013 (3)	0.008 (3)
F4	0.049 (3)	0.093 (4)	0.126 (5)	−0.006 (3)	0.018 (3)	−0.001 (4)
O1	0.095 (4)	0.021 (2)	0.044 (3)	0.013 (3)	0.016 (3)	0.000 (2)
O2	0.124 (5)	0.029 (3)	0.042 (3)	0.000 (3)	0.002 (3)	−0.002 (2)
N1	0.036 (3)	0.027 (3)	0.026 (3)	−0.002 (2)	0.001 (2)	−0.003 (2)
N2	0.040 (3)	0.024 (3)	0.033 (3)	0.000 (2)	0.002 (2)	0.000 (2)
N3	0.037 (3)	0.023 (3)	0.027 (3)	0.001 (2)	0.000 (2)	−0.003 (2)
N4	0.039 (3)	0.025 (3)	0.027 (3)	−0.003 (2)	0.001 (2)	−0.005 (2)
C1	0.039 (4)	0.025 (3)	0.033 (4)	−0.001 (3)	0.004 (3)	0.000 (3)
C2	0.040 (4)	0.028 (3)	0.024 (3)	0.001 (3)	0.007 (3)	0.001 (3)
C3	0.029 (3)	0.028 (3)	0.029 (3)	−0.001 (3)	0.004 (3)	−0.002 (3)
C4	0.058 (5)	0.027 (4)	0.044 (4)	−0.007 (3)	−0.001 (4)	0.000 (3)
C5	0.047 (4)	0.035 (4)	0.032 (4)	−0.005 (3)	0.001 (3)	−0.006 (3)
C6	0.044 (4)	0.035 (4)	0.027 (4)	0.002 (3)	0.004 (3)	0.002 (3)
C7	0.041 (4)	0.020 (3)	0.030 (4)	0.000 (3)	0.002 (3)	0.000 (3)
C8	0.034 (4)	0.027 (3)	0.047 (4)	0.002 (3)	0.001 (3)	−0.004 (3)
C9	0.056 (5)	0.043 (4)	0.031 (4)	−0.006 (3)	−0.011 (3)	0.000 (3)
C10	0.053 (5)	0.029 (4)	0.033 (4)	0.002 (3)	−0.004 (3)	0.005 (3)
C11	0.038 (4)	0.040 (4)	0.045 (4)	−0.001 (3)	0.010 (3)	0.000 (3)
C12	0.036 (4)	0.037 (4)	0.034 (4)	−0.003 (3)	−0.001 (3)	−0.007 (3)
C13	0.036 (4)	0.025 (3)	0.038 (4)	−0.003 (3)	−0.002 (3)	0.000 (3)
C14	0.033 (4)	0.034 (4)	0.030 (4)	−0.003 (3)	0.001 (3)	0.002 (3)
C15	0.040 (4)	0.040 (4)	0.022 (3)	0.004 (3)	0.000 (3)	−0.004 (3)
C16	0.072 (5)	0.032 (4)	0.048 (5)	−0.010 (4)	0.002 (4)	0.005 (3)
C17	0.063 (5)	0.051 (5)	0.030 (4)	0.002 (4)	0.004 (4)	−0.014 (3)
C18	0.053 (4)	0.027 (4)	0.034 (4)	0.009 (3)	0.003 (3)	−0.004 (3)
C19	0.055 (4)	0.021 (3)	0.036 (4)	0.006 (3)	−0.002 (3)	0.003 (3)
C20	0.067 (6)	0.037 (4)	0.052 (5)	−0.003 (4)	−0.008 (4)	0.008 (4)
C21	0.167 (12)	0.046 (5)	0.047 (6)	−0.006 (6)	−0.051 (7)	0.008 (4)
C22	0.231 (18)	0.044 (6)	0.025 (5)	0.015 (8)	0.011 (8)	0.012 (4)
C23	0.147 (12)	0.062 (7)	0.065 (7)	0.016 (7)	0.052 (7)	0.016 (6)
C24	0.072 (6)	0.048 (5)	0.055 (5)	0.000 (4)	0.013 (5)	0.003 (4)

### Geometric parameters (Å, °)

**Table:**

Br1—C2	1.877 (6)	C7—C12	1.389 (9)
Br2—C14	1.881 (6)	C8—C9	1.371 (10)
F1—C8	1.358 (8)	C9—C10	1.375 (10)
F2—C12	1.363 (8)	C9—H9A	0.9500
F3—C20	1.347 (9)	C10—C11	1.370 (10)
F4—C24	1.351 (10)	C10—H10A	0.9500
O1—C6	1.185 (8)	C11—C12	1.368 (9)
O2—C18	1.210 (8)	C11—H11A	0.9500
N1—C3	1.390 (8)	C13—C14	1.416 (9)
N1—N2	1.401 (7)	C13—C16	1.489 (9)
N1—C6	1.403 (8)	C14—C15	1.345 (9)
N2—C1	1.311 (8)	C15—C17	1.487 (9)
N3—N4	1.380 (7)	C16—H16A	0.9800
N3—C15	1.390 (8)	C16—H16B	0.9800
N3—C18	1.403 (8)	C16—H16C	0.9800
N4—C13	1.311 (8)	C17—H17A	0.9800
C1—C2	1.438 (9)	C17—H17B	0.9800
C1—C4	1.491 (8)	C17—H17C	0.9800
C2—C3	1.346 (9)	C18—C19	1.494 (9)
C3—C5	1.491 (8)	C19—C20	1.369 (10)
C4—H4A	0.9800	C19—C24	1.373 (11)
C4—H4B	0.9800	C20—C21	1.371 (12)
C4—H4C	0.9800	C21—C22	1.350 (16)
C5—H5A	0.9800	C21—H21A	0.9500
C5—H5B	0.9800	C22—C23	1.358 (17)
C5—H5C	0.9800	C22—H22A	0.9500
C6—C7	1.506 (8)	C23—C24	1.382 (13)
C7—C8	1.382 (9)	C23—H23A	0.9500
			
C3—N1—N2	112.0 (5)	C10—C11—H11A	121.4
C3—N1—C6	128.7 (5)	F2—C12—C11	119.2 (6)
N2—N1—C6	119.3 (5)	F2—C12—C7	116.7 (6)
C1—N2—N1	104.8 (5)	C11—C12—C7	124.1 (6)
N4—N3—C15	112.0 (5)	N4—C13—C14	110.5 (5)
N4—N3—C18	119.5 (5)	N4—C13—C16	121.2 (6)
C15—N3—C18	128.3 (5)	C14—C13—C16	128.3 (6)
C13—N4—N3	104.9 (5)	C15—C14—C13	108.3 (6)
N2—C1—C2	110.2 (5)	C15—C14—Br2	125.1 (5)
N2—C1—C4	120.9 (6)	C13—C14—Br2	126.6 (5)
C2—C1—C4	128.9 (6)	C14—C15—N3	104.4 (5)
C3—C2—C1	108.3 (5)	C14—C15—C17	130.6 (6)
C3—C2—Br1	125.9 (5)	N3—C15—C17	125.1 (6)
C1—C2—Br1	125.7 (5)	C13—C16—H16A	109.5
C2—C3—N1	104.7 (5)	C13—C16—H16B	109.5
C2—C3—C5	130.8 (6)	H16A—C16—H16B	109.5
N1—C3—C5	124.5 (6)	C13—C16—H16C	109.5
C1—C4—H4A	109.5	H16A—C16—H16C	109.5
C1—C4—H4B	109.5	H16B—C16—H16C	109.5
H4A—C4—H4B	109.5	C15—C17—H17A	109.5
C1—C4—H4C	109.5	C15—C17—H17B	109.5
H4A—C4—H4C	109.5	H17A—C17—H17B	109.5
H4B—C4—H4C	109.5	C15—C17—H17C	109.5
C3—C5—H5A	109.5	H17A—C17—H17C	109.5
C3—C5—H5B	109.5	H17B—C17—H17C	109.5
H5A—C5—H5B	109.5	O2—C18—N3	121.6 (6)
C3—C5—H5C	109.5	O2—C18—C19	123.0 (6)
H5A—C5—H5C	109.5	N3—C18—C19	115.4 (5)
H5B—C5—H5C	109.5	C20—C19—C24	117.6 (7)
O1—C6—N1	121.9 (6)	C20—C19—C18	121.3 (7)
O1—C6—C7	123.2 (6)	C24—C19—C18	121.0 (7)
N1—C6—C7	114.9 (5)	F3—C20—C19	117.2 (7)
C8—C7—C12	115.5 (6)	F3—C20—C21	120.6 (9)
C8—C7—C6	122.2 (6)	C19—C20—C21	122.2 (9)
C12—C7—C6	122.0 (6)	C22—C21—C20	118.1 (10)
F1—C8—C9	120.4 (6)	C22—C21—H21A	120.9
F1—C8—C7	116.8 (6)	C20—C21—H21A	120.9
C9—C8—C7	122.9 (6)	C21—C22—C23	122.5 (10)
C8—C9—C10	118.3 (7)	C21—C22—H22A	118.8
C8—C9—H9A	120.9	C23—C22—H22A	118.8
C10—C9—H9A	120.9	C22—C23—C24	118.1 (11)
C11—C10—C9	122.1 (7)	C22—C23—H23A	120.9
C11—C10—H10A	119.0	C24—C23—H23A	120.9
C9—C10—H10A	119.0	F4—C24—C19	117.4 (8)
C12—C11—C10	117.2 (7)	F4—C24—C23	121.2 (9)
C12—C11—H11A	121.4	C19—C24—C23	121.4 (10)
			
C3—N1—N2—C1	−1.0 (7)	C8—C7—C12—C11	1.0 (10)
C6—N1—N2—C1	177.4 (6)	C6—C7—C12—C11	175.0 (6)
C15—N3—N4—C13	0.5 (7)	N3—N4—C13—C14	−0.1 (7)
C18—N3—N4—C13	176.6 (6)	N3—N4—C13—C16	−179.4 (6)
N1—N2—C1—C2	1.1 (7)	N4—C13—C14—C15	−0.3 (8)
N1—N2—C1—C4	−179.2 (6)	C16—C13—C14—C15	178.9 (7)
N2—C1—C2—C3	−0.9 (8)	N4—C13—C14—Br2	−179.3 (5)
C4—C1—C2—C3	179.4 (7)	C16—C13—C14—Br2	−0.1 (11)
N2—C1—C2—Br1	178.8 (5)	C13—C14—C15—N3	0.6 (7)
C4—C1—C2—Br1	−0.9 (11)	Br2—C14—C15—N3	179.6 (4)
C1—C2—C3—N1	0.3 (7)	C13—C14—C15—C17	−179.6 (7)
Br1—C2—C3—N1	−179.4 (4)	Br2—C14—C15—C17	−0.6 (11)
C1—C2—C3—C5	178.5 (6)	N4—N3—C15—C14	−0.7 (7)
Br1—C2—C3—C5	−1.2 (11)	C18—N3—C15—C14	−176.4 (6)
N2—N1—C3—C2	0.4 (7)	N4—N3—C15—C17	179.5 (6)
C6—N1—C3—C2	−177.8 (6)	C18—N3—C15—C17	3.8 (11)
N2—N1—C3—C5	−177.9 (6)	N4—N3—C18—O2	−175.1 (7)
C6—N1—C3—C5	3.8 (10)	C15—N3—C18—O2	0.3 (11)
C3—N1—C6—O1	10.4 (11)	N4—N3—C18—C19	4.4 (9)
N2—N1—C6—O1	−167.7 (6)	C15—N3—C18—C19	179.8 (6)
C3—N1—C6—C7	−168.9 (6)	O2—C18—C19—C20	−103.7 (9)
N2—N1—C6—C7	13.0 (8)	N3—C18—C19—C20	76.9 (8)
O1—C6—C7—C8	67.7 (10)	O2—C18—C19—C24	72.5 (10)
N1—C6—C7—C8	−113.0 (7)	N3—C18—C19—C24	−107.0 (8)
O1—C6—C7—C12	−105.9 (8)	C24—C19—C20—F3	−178.2 (7)
N1—C6—C7—C12	73.4 (8)	C18—C19—C20—F3	−2.0 (10)
C12—C7—C8—F1	179.5 (6)	C24—C19—C20—C21	1.8 (11)
C6—C7—C8—F1	5.5 (9)	C18—C19—C20—C21	178.1 (7)
C12—C7—C8—C9	−1.1 (9)	F3—C20—C21—C22	179.0 (8)
C6—C7—C8—C9	−175.1 (6)	C19—C20—C21—C22	−1.1 (13)
F1—C8—C9—C10	−178.6 (6)	C20—C21—C22—C23	−0.2 (16)
C7—C8—C9—C10	2.0 (10)	C21—C22—C23—C24	0.6 (16)
C8—C9—C10—C11	−2.8 (10)	C20—C19—C24—F4	179.0 (7)
C9—C10—C11—C12	2.6 (10)	C18—C19—C24—F4	2.7 (11)
C10—C11—C12—F2	−179.8 (6)	C20—C19—C24—C23	−1.3 (12)
C10—C11—C12—C7	−1.7 (10)	C18—C19—C24—C23	−177.6 (8)
C8—C7—C12—F2	179.0 (6)	C22—C23—C24—F4	179.9 (9)
C6—C7—C12—F2	−7.0 (9)	C22—C23—C24—C19	0.2 (14)
